# Intermittent Catheters with Integrated Amphiphilic Surfactant Reduce Urethral Microtrauma in an *Ex Vivo* Model Compared with Polyvinylpyrrolidone-Coated Intermittent Catheters †

**DOI:** 10.3390/jfb16070256

**Published:** 2025-07-10

**Authors:** Luca Barbieri, Makhara S. Ung, Katherine E. Hill, Ased Ali, Laura A. Smith Callahan

**Affiliations:** 1 Convatec Ltd., CTEC Deeside, First Avenue, Deeside Industrial Park, Deeside CH5 2NU, UK; luca.barbieri@convatec.com (L.B.); ased.ali@convatec.com (A.A.); 2 Convatec Ltd., CTEC Boston, 20 Maguire Road, Lexington, MA 02421, USA; makhara.ung@convatec.com (M.S.U.); katherine.hill@convatec.com (K.E.H.)

**Keywords:** catheter, polyvinylpyrrolidone (PVP), amphiphilic surfactant, mucoadhesion, urethra

## Abstract

Intermittent catheterization mitigates urinary retention for over 300,000 people in the US every year, but can cause microtrauma in the urothelium, compromising its barrier function and increasing the risk of pathogen entry, which may affect user health. To reduce adverse effects, intermittent catheters (ICs) with increased lubricity are used. A common strategy to enhance IC lubricity is to apply a polyvinylpyrrolidone (PVP) coating to ICs; however, this coating can become adhesive upon drying, potentially leading to microtrauma. An alternative approach for lubricity is the migration of integrated amphiphilic surfactant (IAS) within the IC to the surface. The present work examines differences in urethral microtrauma caused by the simulated catheterization of *ex vivo* porcine urethral tissue using PVP-coated and IAS ICs. Scanning electron microscopy and fluorescence microscopy of the tissue showed the removal of the apical cell layer after contact with the PVP-coated ICs, but not the IAS IC. More extracellular matrices and DNA were observed on the PVP-coated ICs than the IAS IC after tissue contact. Contact angle analysis of the polar and dispersive components of the surface energy demonstrated that the PVP-coated ICs promoted mucoadhesion, while the IAS IC limited mucoadhesion. Overall, the results indicate that IAS ICs cause less microtrauma to urethral tissue than traditional PVP-coated ICs.

## 1. Introduction

Every year, over 300,000 Americans rely on intermittent catheterization to reduce urinary retention, a condition that leads to permanent bladder damage and kidney disease [1,2,3,4]. Intermittent catheterization has substantial benefits over indwelling catheterization due to a lower risk of urinary tract infections (UTIs), greater patient autonomy, and fewer barriers to intimacy and sexual activity [4,5,6].

In the early 2000s, the introduction of a hydrophilic coating to intermittent catheters (ICs) enhanced their lubricity upon wetting and ease of use [7,8]. Despite its benefits, this technology has limitations based on the material of choice of the coating: polyvinylpyrrolidone (PVP) has a tendency to become adhesive upon drying [9], particularly when in contact with another hydrophilic surface [10]. Since its application in ICs, users reported a ‘sticking’ sensation upon catheter removal [11,12] and contradictory reports are present in the literature in regard to whether it effectively reduces traces of blood in urine (hematuria), compared to uncoated catheters [13,14,15]. Together these reports suggest that PVP-coated ICs adhere to the urethra during bladder voiding, causing microtrauma to the urothelium when the catheter is withdrawn.

The urothelium is a stratified transitional epithelium lining the lower urinary tract, up to the proximal urethra, it constitutes a barrier that protects the underlying tissue from pathogens and toxins present in the urine [16,17,18,19]. An important constituent of this barrier are Glycosaminoglycans (GAGs), which are highly hydrophilic polysaccharides that form a waterproof layer that prevents bacterial adhesion [16,17]. It has been reported that a damaged urothelium can result in pain and increased UTI occurrence [20,21].

An alternative technology to achieve catheter lubricity involves an integrated amphiphilic surfactant (IAS) within the IC material. In this system, the hydrophilic head of the surfactant protrudes from the catheter’s surface upon wetting, creating a highly lubricious water layer. This approach has recently been shown to generate lower adhesion [9] and minimal urothelial cell delamination *in vitro* compared with PVP-coated ICs [22]. Currently, no empirical data are present in the literature that allow us to compare the consequences of the mucoadhesion of these IC technologies to the urethral tissue.

Mucoadhesion is the process by which a material adheres to a mucosal membrane, like the urethra. This multistage process has been extensively studied, from the creation of a close contact between two surfaces to the interpenetration of the polymer and mucosal chains [23,24,25]. Surface energy is thought to have an important role in establishing the initial contact [25,26,27], with the formation of Lewis acid–base bonds, semi-permanent van der Waals interactions, hydrophobic interactions and hydrogen bonding [28,29]. Peppas and Buri [30] modelled this phase of mucoadhesion by mapping the polar and dispersive components of the surface energy of the adhering materials. Lehr et al. [31] found that stronger adhesion between polymers was possible when they displayed similar ratios between the polar and dispersive components. In this work, the polar and dispersive components of the surface energy of the urethral tissue and ICs were evaluated via contact angle techniques. 

Recent literature on ICs has focused on microtrauma to the bladder mucosa in relation to the size of the catheter eyelets [32,33,34]. This work expands on the previous *in vitro* observations [9,22], and utilizes an *ex vivo* porcine model to compare the microtrauma caused to the urethra by PVP-coated and IAS ICs, using imaging and biochemical quantifications.

A controlled test was used to replicate the contact between the tissue and catheter that takes place when the latter is stationary in the urethra during bladder voiding [35,36]. Post-contact, changes in the urothelial tissue surface and transfer of biological material to the ICs were examined, providing insights into how surface properties affect the adhesion to biological tissue.

The data hereby presented was partially published previously in conference papers from the European Association of Urology [37] and the International Continence Society [38]. This publication presents an expanded data set with additional test groups, new images have been included and the biochemistry is assessed from different experiments using an alternative solubilization technique. Furthermore, this work contains surface energy analysis utilized to rationalize the different degrees of microtrauma observed.

## 2. Materials and Methods

### 2.1. Materials

Unless specified, all materials were purchased from Sigma Aldrich (Burlington, MA, USA).

### 2.2. Preparation of Porcine Tissue

*Ex vivo* male porcine urethras were purchased from Lampire Biological laboratories slaughterhouse (Pipersville, PA, USA) and stored at −20 °C [35,39]. Prior to use, urethras were thawed overnight at 4 °C and then at room temperature [35,39]. External glands and connective tissue were removed and the urethras were opened from tip to bladder neck, then dissected into 1 × 3 cm transversal segments. The proximal urethra, approximately the 2 cm of tissue closest to the bladder, was washed in 1× phosphate-buffered saline (PBS), fixed on its external side via adhesive tape onto a 90 mm plastic Petri dish and used for experiments.

### 2.3. Catheters

In-date male ICs with a French size of 12 were obtained from 180 Medical (Oklahoma City, OK, USA). Six products were evaluated: four hydrophilic PVP-coated catheters (Brand A: Coloplast SpeediCath Flex (Humlebaek Denmark), Brand B: Wellspect LoFric Origo (Mölndal, Sweden), Brand C: Hollister VaPro (Libertyville, IL, USA), and Brand D: Coloplast Luja (Humlebaek, Denmark)), and an IAS catheter (ConvaTec GentleCath Glide with FeelClean Technology (London, UK)). All catheters were hydrated according to manufacturer instructions, cut into 1.5 cm long segments immediately prior to testing and mounted onto 3D-printed fixtures that were connected to the Load Cell of a tensile machine for adhesion testing.

### 2.4. Simulated Catheterization Adhesion Testing

A Z005 Universal Tensile Machine (Zwick Roell, Ulm, Germany) equipped with a 10 N Load Cell was used to press the IC to the inner surface of the proximal urethra sections with 3 N of force for 2 min. French size 12 ICs have a diameter of 4 mm [40]. Assuming that only the bottom half of the catheter makes contact with the opened urethral segment, the IC contact area is 0.942 cm^2^ and leads to a contact pressure of 31.83 kPa, which is within the range of pressures generated when opening a urethra with an IC [35]. Regarding the contact time, a recent study [41] assessing 25 patients indicated that the median time required to perform clean intermittent catheterization was 2 min and 23 s. An analysis by Lee et al. [36] focused on the urine flow rate in ICs, taking into account intravesical pressure, viscosity and catheter size. With a 20 cm H_2_O intravesical pressure, the flow of normal urine at 37 °C from a CH12 catheter is 2.714 cc/s. Normal functional bladder capacity in adults ranges from 300 to 400 mL [42]; utilizing a CH12 catheter, this would yield a voiding time ranging from 1.84 to 2.46 min.

### 2.5. Scanning Electron Microscopy (SEM)

Post-adhesion testing, the tissue samples were prepared for SEM as previously described [43]. Briefly, samples were washed with a 0.1 M cacodylate buffer and fixed in 2.5% Glutaraldehyde overnight. The samples were then washed again with the 0.1 M cacodylate buffer and post-fixed for 1 h in 1% Osmium Tetroxide. Next, samples were dehydrated via an ethanol gradient (50%, 70%, 90%, 95%, and 100%) over 3 h and ultimately dried with hexamethyldisilazane (HMDS). Samples were then gold coated using a Manuel Sputter Coater (Agar Scientific, Rotherham, UK) and imaged utilizing a Phenom ProX SEM (Thermofisher, Waltham, MA, USA) at 2000× magnification and a 10 kV accelerating voltage.

### 2.6. Confocal and Fluorescence Imaging

Prior to adhesion testing, urethral tissue samples were stained with 5 μM of DRAQ5 (Thermofisher, Cat. #: 62251) and 5.0 µg/mL of Wheat Germ Agglutin (WGA) conjugated with Alexa Fluor 555 (Thermofisher, Cat. #: W32464) in PBS for 20 min at room temperature in dark conditions. The samples were then washed three times in 1× PBS to discard excess dye and allowed to dry in dark conditions at room temperature for 15 min before adhesion testing.

Post contact with the tissue, IC samples were allowed to dry in the dark for 2 h before imaging. For confocal imaging, IC samples were mounted on a 35 mm glass-bottom dish with 50 μL of Fluoromount-G Mounting Medium (ThermoFisher, Waltham, MA, USA, Cat. #: 00-4958-02). Z-Stacks of the samples were taken on a SP8 confocal microscope (Leica Microsystems, Deerfield, IL, USA) at 10× magnification. ImageJ software v. 1.54 was used to black balance image background intensity. For fluorescent imaging, ICs and urethral tissue were fixed in 4% paraformaldehyde (ThermoFisher, Waltham, MA, USA, Cat. #: J61899.AK) overnight. ICs were cut into 15 μm cross-sections and urethral tissue was cut into 10 μm cross-sections using a Cryo3 cryostat equipped with a low-profile blade (Sakura, Torrance, CA, USA). Fluorescent images were taken on a BZ-800 fluorescent microscope (Keyence, Itasca, IL, USA) at 20× magnification. ImageJ software was used to black balance image background intensity and the images were cropped for size.

### 2.7. Alcian Blue Staining

IC samples were dried at room temperature for 2 h, then fixed overnight in 10% neutral buffered formalin solution. Samples were then stained with Alcian Blue, similar to the method previously described [44]. Briefly, each sample was transferred in a 3% acetic acid solution for 20 min, followed by 3 h of staining in 1% Alcian blue, at a pH of 2.5, at room temperature. The samples were then washed three times in 1× PBS for 20 min to disperse the excess dye. Samples were then mounted in 35 × 10 mm plastic Petri dishes with 50 μL of Fluoromount-G Mounting medium and Z-Stacks were acquired using a BZ-800 microscope in brightfield mode. Post-acquisition, the images were white balanced in ImageJ software.

### 2.8. Biochemical Quantifications

ICs post-urethra contact were incubated in a 50 μg/mL Protease K solution for 2.5 h at 55 °C, followed by heat shock at 90 °C for 10 min to inactivate the enzyme. Samples were centrifuged for 10 min at 10,000 rpm to pellet undigested material. For the quantification of DNA, 2 μL aliquots of the digest were placed on a NanoQuant plate and read on a Tecan Spark (Morgan Hill, CA, USA) plate reader.

The sulfated glycosaminoglycan (sGAG) content was quantified via a Dimethyl Methylene Blue kit (Chondrex, Woodinville, WA, USA, Cat. #: 6022) according to manufacturer instructions. Dye solution was added to the digest in a 96-well plate and the absorbance was read at 525 nm on a Tecan Spark plate reader.

### 2.9. Contact Angle Measurements

Contact angle measurements of the tested IC products and urethral tissue were carried out in captive bubble mode [45] using an Advanced DSA 25 (KRUSS, Matthews, NC, USA). To create a flat testing surface, 2.5 cm long IC segments without eyelets were cut longitudinally, flattened and fixed to laminated paper. Proximal urethra tissue samples were washed in MilliQ water then fixed to laminated paper. The samples were then submerged, facing down, in a cuvette containing room-temperature MilliQ water. A J-shaped needle connected to a glass syringe was used to dispense 4 μL drops of air or n-octane to the surface of the sample. The drops were allowed to equilibrate onto the sample’s surface for 30 s before an image was collected. Following image acquisition, the contact angle between the sample surface and the drop was calculated using KRUSS Advance software.

### 2.10. Surface Energy Calculations

The surface energy of the IC products and the urethra tissue was investigated by implementing the mathematical work of Roudman et al. [46], who applied the Owens and Wendt [47] refinements to the Fowkes [48] method. The resulting Equations (1) and (2) allow for the calculation of the polar ($\gamma_{s}^{p})\;$and dispersive ($\gamma_{s}^{d})\;$surface energy components of the tested surfaces from the measured angles between the sample and an air ($\theta_{a})\;$or n-octane ($\theta_{o})$ bubble.

(1) $$\gamma_{s}^{d}=\frac{{\left[\gamma_{wv}\left(1+\cos\theta_{a}\right)-2\sqrt{50.8\;\gamma_{s}^{p}}\right]}^{2}}{4\gamma_{wv}^{d}}=2.3307\;[5.093\;{\left(1+cos\theta_{a})-\sqrt{\gamma_{s}^{p}}\right]}^{2}$$

(2) $$\gamma_{s}^{p}=\frac{{\left(\gamma_{wv}-\gamma_{ov}+\gamma_{wv}cos\theta_{o}\right)}^{2}}{4\gamma_{s}^{d}}=12.7\;{\left(1+cos\theta_{o}\right)}^{2}$$

The subscripts *s*, *w* and *a* refer to the phase (sample, water and air, respectively) and the superscripts *d* and *p* indicate the dispersive and polar components of the surface energy. The surface free energies of interaction between water or octane and their vapours (v) are reported as $\gamma_{wv}=$ 72.6 dyn/cm and $\gamma_{ov}=$ 21.8 dyn/cm [46].

### 2.11. Statistical Analysis

For imaging studies, three biological replicates were performed. For biochemical quantifications, two independent experiments were conducted with three replicates per group per experiment. For contact angle experiments, 10 catheter samples per test group and 10 sections per urethra (N = 3 biological replicates) were analyzed. All quantified data is reported as the mean and standard deviation. Statistical analysis was performed using GraphPad Prism 10.4.0 for Windows (GraphPad Software, Boston, MA, USA). ANOVA tests were conducted where appropriate to determine significance. Significance was set as a *p*-value of less than 0.05.

## 3. Results

### 3.1. Imaging the Urothelial Tissue After IC Contact

**Figure 1:**
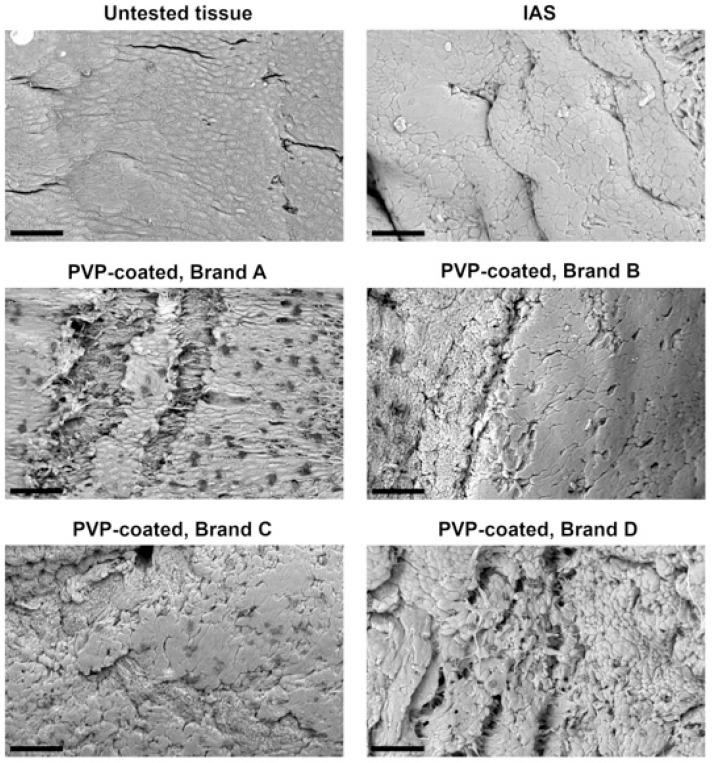
Scanning electron microscope (SEM) micrographs of pristine urethral tissue (top left) and tissue following 2 min of contact with integrated amphiphilic surfactant (IAS) and polyvinylpyrrolidone (PVP)-coated intermittent catheter products. Three biological replicates were performed. Scale bars are 40 μm. Image of the tissue post-contact with PVP-coated Brand A was previously published in a conference paper from the European Association of Urology [37].

Following a single 2 min contact with IC samples, the damage to the urothelium was visualized via SEM. Figure 1 includes a micrograph of the control urothelial tissue, displaying a compact and uniform apical cell layer of superficial polygonal-shaped cells, similar to previous studies [49,50]. Post-contact with the IAS IC, the cell layer appears to be more compact than in the control. Post-contact with the PVP-coated ICs, different levels of damage can be observed, with Brand A producing visible delamination of the apical layer, Brand B and C dislocating single cells or cell aggregates and Brand D creating an amorphous and irregular tissue surface.

**Figure 2:**
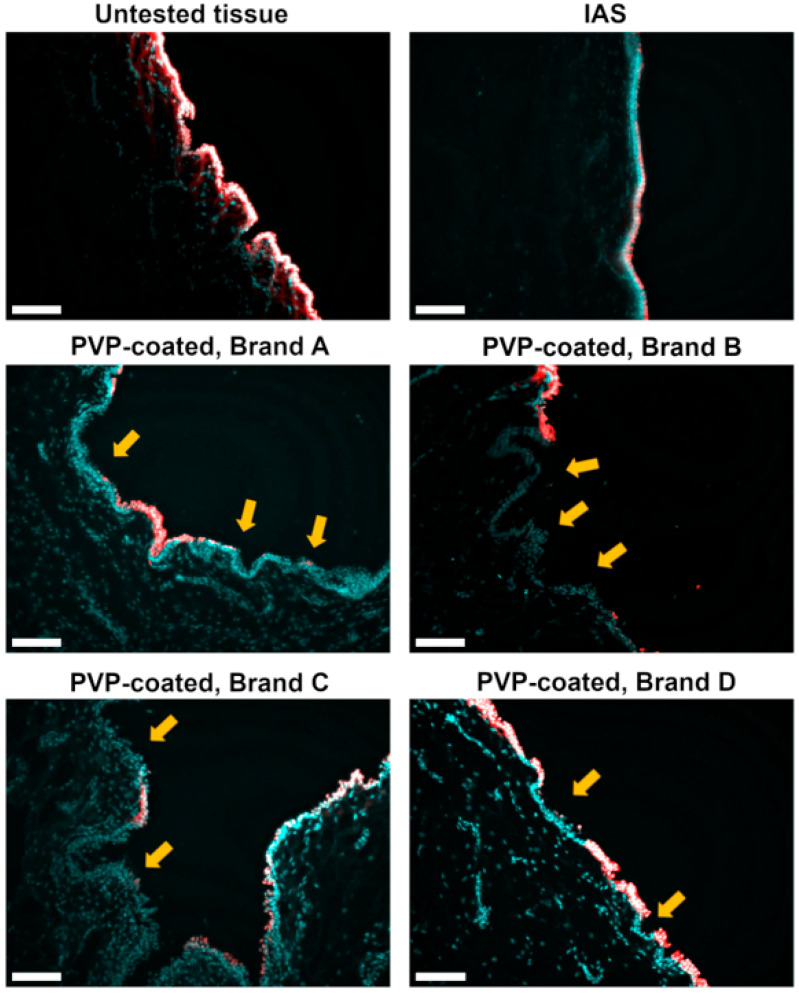
Sections of untested urethral tissue (top left) and tissue following 2 min of contact with integrated amphiphilic surfactant (IAS) and polyvinylpyrrolidone (PVP)-coated intermittent catheter products. Tissue samples were stained with DRAQ5 (cyan) for nuclear and wheat germ agglutinin (red) for cellular membrane visualization. The yellow arrows indicate areas of damage. The presence of intact cells is detected by white fluorescence. Three biological replicates were performed. Scale bars are 100 μm.

Urothelial damage was also visualized using cell membrane (WGA-red) and nuclear (DRAQ5-cyan) staining of tissue cross-sections (Figure 2). Intact cells appear as white due to the colocalization of cell membrane and nuclear staining. The untested control tissue presents an intact and uniform apical cell layer with an undulated morphology similar to previous reports [51,52]. After contact with the IAS catheter, the tissue appears to be flattened, consistent with the morphology observed via SEM. The tissue sections following contact with all PVP-coated brands show a delamination of the cell lining. In some areas, highlighted by the yellow arrows and particularly evident in Brand C, the apical cells appear to be “lifted” from the ones below, indicating the loss of cohesion between cell layers.

### 3.2. Imaging the IC Samples After Contact with Urethral Tissue

**Figure 3:**
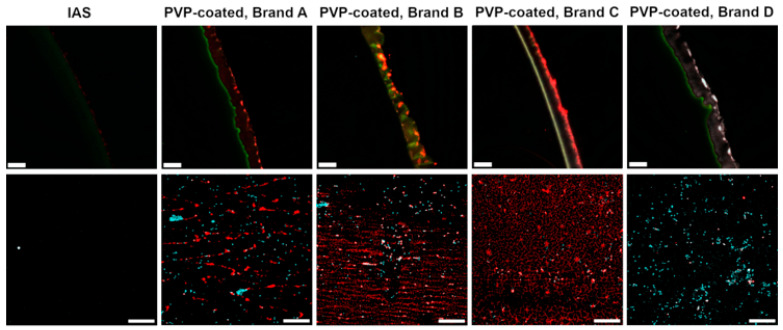
Fluorescent images of catheter cross-sections (**top row**) and confocal microscopy images of catheter surfaces (**bottom row**) after 2 min of contact with *ex vivo* urethras show selectively stained biological transfer: cell nuclei stained with DRAQ5 appear in cyan, cell membranes and the extracellular matrix stained with WGA appear in red, the catheter material shows green self-fluorescence, and white fluorescence indicates the presence of intact cells. Three biological replicates were performed. Scale bars are 50 µm for the top row and 200 µm for the bottom row. Image of the cross-section of PVP-coated Brand D post-contact with urethral tissue was previously published in a conference paper from the International Continence Society [38].

Biological transfer from urethral tissue to the IC samples was imaged via fluorescent and confocal microscopy, cell nuclei stained with DRAQ5 appear in cyan, cell membranes and the extracellular matrix (ECM) stained via WGA appear in red, the co-localization of nuclear and cellular membrane staining appear in white. The top row of Figure 3 shows IC cross-sections where the catheter material is visible in green, while a PVP layer, absent in the IAS catheter, shows an opaque appearance. Confocal images in the bottom row of Figure 3 display surfaces of ICs post-contact with urethral tissue. Less biological material was observed on the IAS IC than on the PVP-coated ICs (Figure 3). Brands A and B present multiple clusters of cells and WGA-stained ECM oriented with the topography of the coating surface. Brand C shows a lower concentration of cells compared with the other PVP-coated ICs, but a higher ECM transfer. Brand D presents the highest concentration of cells at its surface, imaged also in the cross-section as white clusters on the PVP coating.

**Figure 4:**
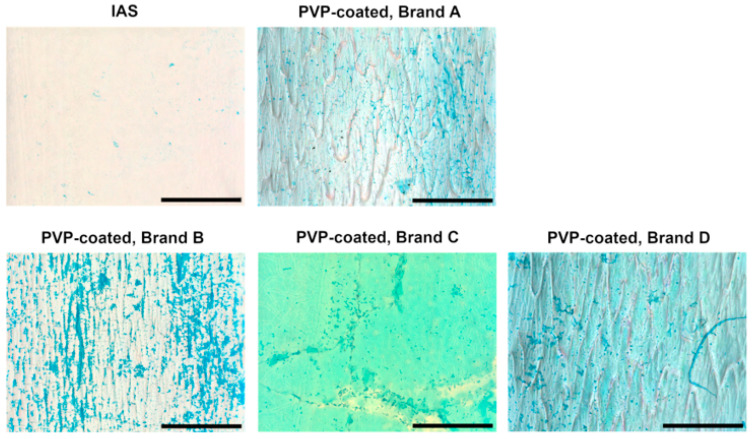
Brightfield images of integrated amphiphilic surfactant (IAS) and polyvinylpyrrolidone (PVP)-coated intermittent catheter surfaces following 2 min of contact with porcine urethras and consequent Alcian Blue staining. Glycosaminoglycans (GAGs) are visible in a darker blue shade compared to the catheters’ surface. Three biological replicates were performed. Scale bars are 500 μm.

Glycosaminoglycans (GAGs) are part of the impermeable lining of the lower urinary tract that protects the underlying cells from any toxic substance in the urine and from bacterial attachment [16,53]. GAG transfer from the urethral tissue to the ICs was examined using Alcian Blue staining and imaged via light microscopy (Figure 4). Small traces of the Alcian Blue-stained GAGs are visible on the surface of the IAS IC, while most PVP-coated samples show a distinctively higher concentration, in a darker shade compared to the light blue appearance of the PVP coating post-Alcian Blue staining. Brand C displays a more opaque background compared to the other PVP-coated ICs; the GAGs transferred onto its surface are aggregated in the proximity of the surface defects, in lower numbers than on the other PVP-coated ICs.

### 3.3. Biochemical Quantification of Transfer from Urethral Tissue to ICs

**Figure 5:**
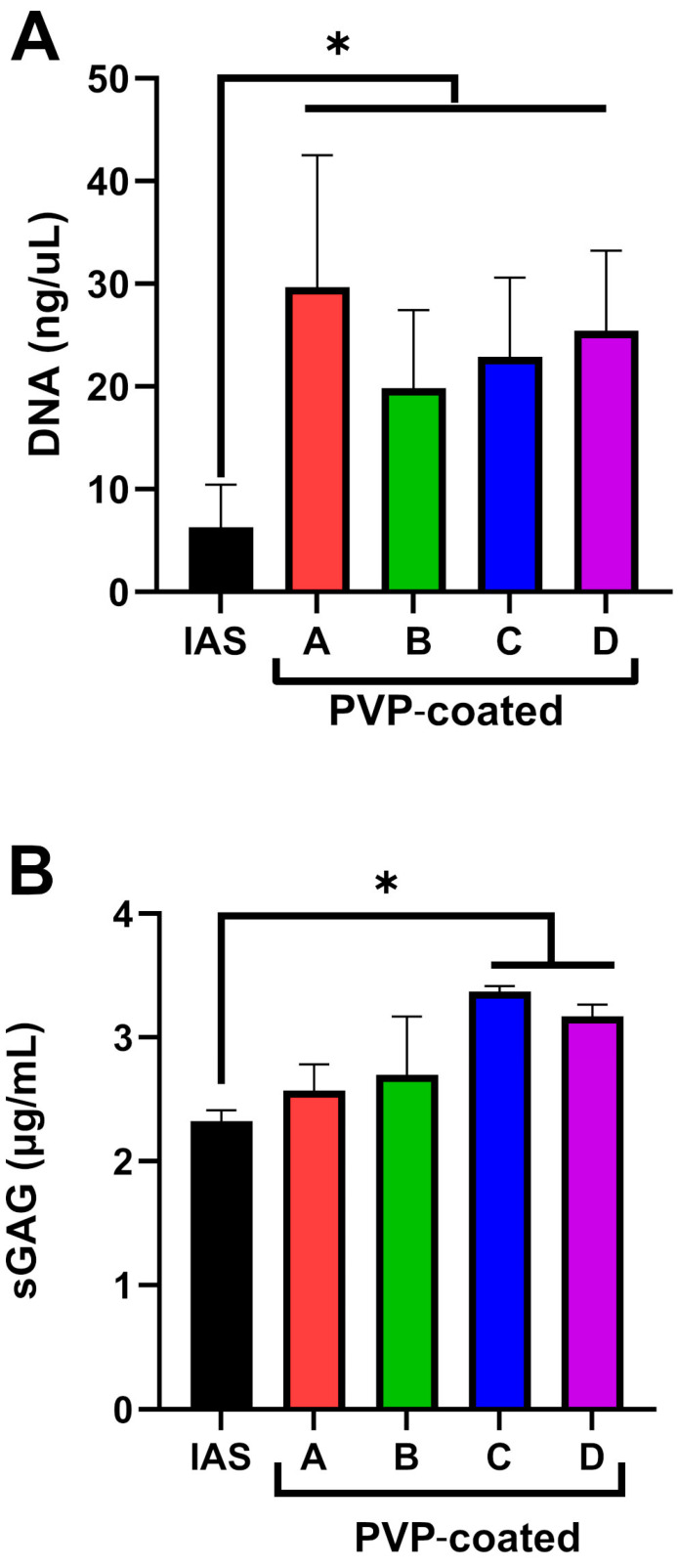
Graphs summarizing the quantification of (**A**) DNA and (**B**) sulfated Glycosaminoglycans (sGAGs) transferred to integrated amphiphilic surfactant (IAS) and polyvinylpyrrolidone (PVP)-coated intermittent catheter samples following 2 min of contact with urethral tissue. Two independent experiments were conducted with three replicates per group per experiment; * indicates *p* ≤ 0.05.

To support imaging studies, biochemical quantifications of DNA (Figure 5A) and GAGs (Figure 5B) on the ICs after urothelial tissue contact were conducted. The data confirm a higher transfer of cells onto the surface of PVP-coated catheters than onto the IAS one, with a significantly lower amount of DNA adhering to the IAS IC compared to the PVP-coated ICs (Figure 5A). No significant difference was found between PVP-coated brands. GAGs quantification (Figure 5B) indicated a lower amount of transfer to the IAS IC than the PVP-coated ICs. Brand C and D ICs were found to have significantly more GAGs than the IAS IC.

### 3.4. Urethral Tissue and ICs Surface Energy Components

The measured contact angles and the surface energy components calculated via Equations (1) and (2) are summarized in Table 1.

The data show clear differences in the polar and dispersive components of the two catheter technologies, with the PVP-coated ICs showing a polar/dispersive ratio that is comparable with the one of the urethral tissue, while a distinctly dispersive surface chemistry characterizes the IAS IC.

## 4. Discussion

Although hydrophilic PVP-coated ICs improve ease of use and comfort [54], they do not resolve hematuria and urethral microtrauma in all users [55,56]. The increase in PVP adhesion upon drying may contribute to these issues [9,10]. Alternative strategies are needed to achieve IC lubricity without compromising the urethral health of the users [57].

Expanding on previous *in vitro* work [9,22], this study examines the microtrauma to *ex vivo* urethral tissue caused by a single IC interaction. Most IC users catheterize four to six times a day [2,58], so cumulative urethral microtrauma is likely greater than observed in this study. Additionally, this study utilizes a controlled adhesion test to model the contact between the urethra and IC which occurs during bladder voiding. Two minutes represents an average IC indwelling time that is common in *in vitro* experiments [9,22,36,59]. In clinical practice, catheterization times are user dependent and vary greatly [41,60,61]; this study may therefore under-represent the IC indwelling time in many users. The adhesion test used in this work does not address the contribution of the shear forces applied by the IC on the urethra during insertion/withdrawal; this contribution is likely minimal due to the extremely low coefficient of friction of hydrophilic ICs [8,9]. As observed in Figure 1 and Figure 2, adhesion forces alone lead to significant microtrauma of urethral tissue.

In this study, variations in PVP coating between brands led to differences in urethral microtrauma after contact. Brand A delaminated portions of the apical urothelial cell layer (Figure 1 and Figure 2). Single cells were missing from the apical urothelial layer after contact with Brand B (Figure 1). Brand C left a morphology resembling interstitial cystitis of the urothelium (Figure 1) [62,63], while the tissue post-contact with Brand D showed a dislocation of cells and an amorphous surface (Figure 1 and Figure 2). The delamination of the apical cell layers, visible in the cross-sections of the tissue (Figure 2) after contact with PVP coatings, was consistent with observations from an *in vivo* rabbit model evaluating the impact of catheterization [64]. This damage to the urothelium likely compromises its barrier function, leaving the underlying cell layers vulnerable to harmful urinary products and uropathogens [16]. In contrast, after contact with the IAS IC, the urothelium appeared to retain its integrity (Figure 1 and Figure 2). Cell transfer (Figure 3) to the ICs was congruent with the microtrauma observed in urethral tissue (Figure 1 and Figure 2). Biochemical quantification of DNA (Figure 5A) further supported the significantly greater transfer of biological material to PVP-coated ICs than to the IAS IC.

GAGs are water-binding molecules on the surface of the urothelium that are important for maintaining its barrier function due to their anti-adherence properties [16,21]. More GAG content was observed on the PVP-coated ICs than on the IAS IC (Figure 4 and Figure 5B) after the adhesion test. Brand C and D ICs were found to have significantly more GAGs than the IAS IC (Figure 5B), further supporting the potential compromise of tissue barrier function [16,21]. These findings have real-world impact for IC users, as microtrauma to the urethral tissue and damage to the GAG layer have been reported to increase the occurrence of UTIs [65,66].

To understand the differences in the observed microtrauma, the contribution of mucoadhesion was investigated. The first stage of mucoadhesion occurs when two surfaces establish intimate contact [23]. Previous studies [25,26,27] suggest that initial contact is governed by surface energy effects, with an important contribution of van der Waals forces [28,29]. Lehr et al. found higher potential for adhesion when the two surfaces have similar ratios between polar and dispersive components [31]. Our findings show that the *ex vivo* porcine urethral tissue possesses a ratio of polar and dispersive surface energy components that is similar to the PVP-coated ICs (Table 1). The approximately two-times-greater polar surface energy component compared with the dispersive component for PVP-coated ICs indicates a high concentration of permanent dipoles at the interface with a propensity to generate attractive Keesom forces with similar surfaces [67], further supporting the potential mucoadhesion between the PVP-coated ICs and urethral tissue. The mostly dispersive surface energy of the IAS IC was notably different from the urethral tissue. Initial adhesion between the IAS IC and the tissue is therefore likely to be driven mostly by relatively weak Debye and London forces [67,68], reducing the potential for mucoadhesion. Overall, the analysis of the polar and dispersive components of the surface energy supports the hypothesis that potential mucoadhesion between the IC and urethral tissue contributes to the observed microtrauma in this study.

### Study Limitations and Future Work

The data presented is related to a single simulated catheterization. Future work should focus on evaluating the damage after multiple catheterizations to understand the long-term impact on urothelial health. Due to the limitations of *ex vivo* tissue, these studies should be carried out utilizing either an advanced *in vitro* model or an animal one, which would allow for the assessment of how tissue repair and the immune system help prevent UTIs post-disruption of the urothelial barrier. Future work should look to deepen the focus on the material chemistry of ICs and how it affects the adhesion to the urethral mucosa; the investigation of acid–base surface energy components and the interpenetration between the ICs’ polymeric chains and the urethral tissue [24] could provide additional insights into how ICs interact with the urethral tissue to cause microtrauma.

## 5. Conclusions

The effects of two hydrophilic IC technologies, PVP coating and IAS, on urethral tissue microtrauma were examined via a controlled test that simulates the contact between the urethra and an IC that occurs during bladder voiding. PVP-coated ICs were found to cause greater damage to the urothelium than the IAS IC. After contact with the tissue, more biological material was found on PVP-coated ICs than the IAS IC, with significantly more DNA found on PVP-coated ICs than the IAS IC. Evaluation of the polar and dispersive components of the surface energy supports the greater mucoadhesive potential between PVP-coated ICs and urethral tissue than the IAS IC and urethral tissue. Taken together, the data indicate that the IAS IC causes less disruption to the urothelium than the more common PVP-coated ICs in an *ex vivo* porcine model. The development of alternative approaches to IC lubricity other than PVP coating could significantly improve IC-user experience and urethral health.

## Figures and Tables

**Table 1:** Measured contact angle values and calculated surface energy components ^1^.

	$\theta_{a}$	$\theta_{o}$	$\gamma_{s}^{d}$ (dyn/cm)	$\gamma_{s}^{p}$ (dyn/cm)
**Urethral Tissue**	38.40° (±0.88)	43.49° (±6.63)	20.39 (±4.45)	37.73 (±3.52)
**IAS**	34.82° (±0.60)	135.63° (±11.72)	158.09 (±20.85)	1.29 (±1.29)
**PVP-coated, Brand A**	29.22° (±1.32)	38.33° (±3.75)	23.66 (±2.96)	40.40 (±1.82)
**PVP-coated, Brand B**	32.08° (±0.15)	36.50° (±3.04)	20.76 (±1.69)	41.30 (±1.77)
**PVP-coated, Brand C**	27.81° (±4.26)	31.38° (±2.12)	20.79 (±1.91)	43.62 (±0.90)
**PVP-coated, Brand D**	32.63° (±2.97)	41.41° (±3.71)	23.18 (±3.96)	38.86 (±1.90)

^1^ $\theta_{a}\;$, $\theta_{o}\;$= measured contact angle values between the tested surfaces and an air bubble or n-octane bubble, respectively. $\gamma_{s}^{d}$, $\gamma_{s}^{p}$ = calculated dispersive and polar components, respectively, of the surface energy of the tested surfaces. Ten catheter samples per test group and 10 sections per urethra (N = 3 biological replicates) were analyzed. Values are indicated as averages (±standard deviation).

## Data Availability

The original contributions presented in the study are included in the article. Further inquiries can be directed to the corresponding author.

## Abbreviations

The following abbreviations are used in this manuscript:

ECM	Extracellular Matrix
GAG(s)	Glycosaminoglycan(s)
HMDS	Hexamethyldisilazane
IAS	Integrated Amphiphilic Surfactant
IC(s)	Intermittent Catheter(s)
PBS	Phosphate-Buffered Saline
PVP	Polyvinylpyrrolidone
SEM	Scanning Electron Microscopy
sGAG	Sulfated Glycosaminoglycan
UTIs	Urinary tract infections
WGA	Wheat Germ Agglutin
